# Intraoperative Adverse Events in Robot-Assisted Minimally Invasive Esophagectomy (RAMIE): Incidence, Management, and Impact on Surgical Outcomes

**DOI:** 10.1097/SLA.0000000000007167

**Published:** 2026-08-19

**Authors:** Franco Badaloni, Adam Zeyara, Gino M. Kuiper, Stijn Vanstraelen, Ludovico Campi, Jan Willem van den Berg, Richard van Hillegersberg, Jelle P. Ruurda

**Affiliations:** 1 Department of Surgery, University Medical Center Utrecht, Utrecht, The Netherlands; 2 Department of Surgery, Skåne University Hospital, Lund, Sweden; 3 Department of Thoracic Surgery, University Hospitals Leuven, Leuven, Belgium; 4 Surgical Science Department, University of Turin, Turin, Italy

**Keywords:** intraoperative adverse events, robotic surgery, esophagectomy, esophageal cancer, intraoperative complications

## Abstract

**Objective:**

Assess the incidence and management of intraoperative adverse events (IAEs) during robot-assisted minimally invasive esophagectomy (RAMIE) and evaluate their impact on postoperative outcomes.

**Background:**

IAEs are an inherent component of surgical practice and owing to its complexity, esophagectomy is no exception. Data on their incidence and management remain limited. Robotic assistance may facilitate management of these events.

**Methods:**

This retrospective observational cohort study analyzed prospectively collected data from consecutive RAMIE performed at UMC Utrecht using the da Vinci Xi system. iAEs were categorized using the ClassIntra classification. Perioperative outcomes were compared according to major IAEs occurrence (ClassIntra ≥III).

**Results:**

Between 2018 and 2025, 478 RAMIE were performed. IAEs occurred in 41 patients (8.6%; 46 events), of which 92.7% were managed robotically without conversion. Bleeding was the most common event (69.6%), followed by chyle leaks (8.7%), lung injuries (8.7%), and airway injuries (4.3%). Major IAEs occurred in 20 patients (4.2%) and were associated with increased mean blood loss (1211 vs. 296 mL, *P*<0.001), higher conversion rates (15.0% vs. 1.3%, *P*=0.005) and longer mean operative time (478.6 vs. 413.3 min, *P*=0.022). Major postoperative complications (35.0% vs. 37.3%, *P*=1.000), median hospital length of stay (11 vs. 11 d, *P*=0.311) and in-hospital mortality (5.0% vs. 2.7%, *P*=0.446) were comparable between groups.

**Conclusion:**

In this retrospective cohort, over 90% of IAEs during RAMIE were managed robotically without conversion, with no significant increase in postoperative morbidity. These results should be interpreted in the context of an expert center and a limited number of events.

Intraoperative adverse events (IAEs) are an inherent component of surgical practice and represent deviations from the ideal operative course.^1^ They are not isolated technical incidents but clinically relevant events, independently associated with increased postoperative morbidity, prolonged length of stay, and higher mortality.^1,2^ However, they remain inconsistently reported, frequently embedded within composite perioperative outcomes and described using heterogeneous definitions.^3^

In this context, esophagectomy, one of the most complex procedures in oncologic surgery, requiring extensive dissection in anatomically confined spaces and proximity to vital structures, is inherently prone to IAEs.^4,5^ While postoperative complications after esophagectomy have been extensively studied, data on these events remain limited across surgical approaches.^3,6,7^ This gap is particularly relevant in robot-assisted minimally invasive esophagectomy (RAMIE), a technique with increasing global adoption and distinct technical advantages such as enhanced dexterity, tremor filtration and stable high-definition three-dimensional visualization that may influence both the occurrence and management of such events.^8–10^

Recent advances now enable more accurate and standardized reporting of IAEs. Video capturing systems within robotic platforms facilitate comprehensive data collection,^11^ while the ClassIntra classification provides a validated and reproducible framework,^1^ capturing a broader spectrum of events than other classifications^12,13^ and demonstrating strong predictive value for postoperative outcomes with excellent inter-rater agreement, supporting its use in complex procedures such as RAMIE.^14,15^

Given the recognized clinical impact of IAEs, the complexity of esophagectomy, and the limited evaluation of these events in robotic surgery, a comprehensive assessment during RAMIE is needed.

Therefore, the aim of this study was to assess the incidence, severity, and management of intraoperative adverse events during robot-assisted minimally invasive esophagectomy, and to evaluate their impact on postoperative outcomes.

## METHODS

This retrospective observational cohort study included all consecutive patients who underwent RAMIE for esophageal cancer at University Medical Center Utrecht, using a four-arm da Vinci Xi system (Intuitive Surgical Inc., Sunnyvale, CA), between May 1, 2018, and October 5, 2025.

Both transthoracic and transhiatal esophagectomies were included, with no exclusion criteria. Data were collected from a prospectively maintained database. The manuscript was reported in accordance with the STROBE statement.^16^

### Assessment and Classification of Intraoperative Adverse Events

IAEs were recorded by the surgical and anesthesiology teams during the surgical sign-out and subsequently reviewed in a weekly multidisciplinary team meeting. Anesthesia-related IAEs were not included in the present analysis, which focused on surgery-related events.

All cases registered with IAEs were identified and independently evaluated and classified by 3 surgeons (FB, AZ, JR) through operative report review and surgical video analysis using the Clinical Assistant software (NEXUS B.V., Vianen, The Netherlands).

IAEs severity was graded using the validated ClassIntra classification, which defines any deviation from the ideal intraoperative course occurring between skin incision and skin closure.^1^ ClassIntra grades range from grade 0, indicating no deviation, to grades I–II requiring no or minor intervention, grades III–IV requiring moderate or major, potentially life-threatening interventions, and grade V corresponding to intraoperative death (see Table 1, Supplemental Digital Content 1, https://links.lww.com/SLA/F917). Major IAEs were defined as ClassIntra grade III or higher.

### Outcome Measures

The primary outcome was the incidence of IAEs. Secondary outcomes included event severity and management, as well as perioperative outcomes: blood loss, operative time, conversion, major postoperative complications (Clavien-Dindo grade ≥III),^17^ ICU stay, hospital length of stay, in-hospital mortality, R0 resection, and lymph node yield. Postoperative outcomes were compared stratified by the occurrence of major IAEs.

Robotic management was defined as complete intraoperative control of IAEs using the robotic platform without undocking or creation of an auxiliary incision. Conversion was defined as undocking of the robotic system with transition to open or conventional minimally invasive surgery.

### Surgical Procedure and Postoperative Management

All procedures were performed by experienced surgeons in robotic upper gastrointestinal surgery (JWvdB, RvH, JR), using a standardized surgical technique.^18–20^ The abdominal phase comprised truncal lymphadenectomy along the common hepatic, left gastric, and splenic arteries, followed by gastric tube formation and jejunostomy placement.^20^ The thoracic phase included systematic mediastinal lymphadenectomy of paratracheal, tracheobronchial, aortopulmonary window and subcarinal stations;^19^ in transhiatal cases, mediastinal dissection was instead performed via the hiatus without thoracic entry. Esophagogastric reconstruction was performed with either intrathoracic or cervical anastomosis. Bilateral chest drains were routinely placed. Enteral nutrition was initiated via jejunostomy on postoperative day 1, with gradual reintroduction of oral intake from day 5.

### Statistical Analysis

Continuous variables were analyzed with the Mann-Whitney *U* test and categorical variables with the χ^2^ or Fisher exact test, as appropriate. Missing data were limited to baseline characteristics and were completed through review of individual patient records.

Univariate and multivariable logistic regression analyses were performed to assess the association between major IAEs and major postoperative complications. The multivariate model was adjusted a priori for ASA score (≥3) and advanced tumor stage (T3 or higher and/or nodal positivity), based on clinical relevance and limited to preserve model stability given the small number of major IAEs. Additionally, Firth-penalized logistic regression was performed as a sensitivity analysis. Odds ratios are reported with 95% confidence intervals. Two-sided *P* values <0.05 were considered significant. Analyses were conducted in Python (version 3.12).

## RESULTS

Among 478 RAMIE procedures, 46 IAEs occurred in 41 patients (8.6%), of whom 20 patients (4.2%) experienced major events (ClassIntra ≥III). The patient flow is illustrated in (Supplementary Figure 1, Supplemental Digital Content 2, https://links.lww.com/SLA/F982).

Baseline characteristics were comparable across all groups Supplementary Table 2, Supplemental Digital Content 4, https://links.lww.com/SLA/F983. Most patients had ASA score II–III (90.8%), and presented with cT3 or higher and/or node-positive disease (81.5%).

A detailed description of IAEs, including ClassIntra severity grade, surgical step, management strategy, corresponding postoperative complication, and whether the complication was considered directly related to the intraoperative event, is provided in Table 1.

**TABLE 1:** Intraoperative Adverse Events: Type, Surgical Step, Management, ClassIntra Grade, and Postoperative Complication Grade

Type	Surgical Step(s)	Management	ClassIntra	Clavien-Dindo
*Bleeding (n=32, 69.6%)*				
Aorto-esophageal branch (n = 11)	Para-aortic dissection Aortic arch dissection	Compression + vessel sealer compression + veriset ± flow sealant	II (n = 2) III (n=7) IV (n=2)	— CD IIIb (n=1) CD IV (n=1)
Azygos vein (n = 5)	Mediastinal pleura dissection Para-aortic dissection	Hem-o-lok suture 4-0 prolene	II (n = 4) III (n=1)	CD IV (n=2) CD IV (n=1)
Hepatic artery (n = 3)	Hepatic artery lymphadenectomy	4-0/5-0 prolene suture	III (n = 3)	—
Left gastric artery (n = 2)	Division of left gastric Left gastric dissection	Vessel sealer **Conversion** + suture prolene 4-0	II (n = 1) III (n = 1)	— —
Subcarinal (n = 2)	Subcarinal lymphadenectomy	Compression compression + vessel sealer	I (n=1) II (n=1)	— —
Splenic artery (n=2)	Splenic artery lymphadenectomy	Suture prolene 4-0	II (n = 1) III (n = 1)	— —
Inferior vena cava (n = 1)	Hepatic artery lymphadenectomy	**Conversion +** suture prolene 4-0	IV	—
Subclavian artery (n = 1)	Lung adhesiolysis	Clip	III	CD IIIb
Right gastric artery (n = 1)	Lesser curvature mobilization	Vessel sealer	II	—
Right bronchial artery (n = 1)	Para-aortic dissection	Vessel sealer	II	—
Aortopulmonary Window (n = 1)	Aortopulmonary lymphadenectomy	Vessel sealer	II	CD IIIb
Right paratracheal (n = 1)	Paratracheal lymphadenectomy	Vessel sealer	I	CD IIIb
Transverse mesocolon (n = 1)	Gastrocolic ligament division	Vessel sealer	I	—
*Chyle leak (n=4, 8.7%)*				
Thoracic duct injury (n = 4)	Thoracic duct dissection Para-aortic dissection	Suture prolene 4-0 Suture prolene 4-0 + clip	II (n = 4)	CD IV* (n=1) CD V (n=2)
*Lung injury (n=4, 8.7%)*				
Parenchymal tear (n = 4)	Adhesiolysis Esophageal mobilization Mediastinal pleura dissection	Suture PDS 4-0	II (n = 4)	—
*Airway injury (n=2, 4.3%)*				
Left main bronchus injury (n = 1)	Tracheoesophageal dissection	Suture prolene 5-0	III	CD IV
Left main bronchus + trachea (n = 1)	Endotracheal tube cuff (intubation)	Tube exchange + suture PDS 5-0	IV	CD V*
*Other injuries (n=4, 8.7%)*				
Pericardial injury (n = 1)	Pericardial dissection	Suture Vicryl 3-0	II	CD IV
Splenic upper pole injury (n = 1)	Hiatal exposure	**Conversion** + splenectomy	IV	—
Diaphragmatic defect (n = 1)	Trocar placement (10th intercostal space)	Suture Vicryl 1-0	III	CD IV
Colonic serosal injury (n = 1)	Gastrocolic ligament division	Suture Vicryl 3-0	II	CD IIIa

* Postoperative complication considered directly related to the intraoperative adverse event.

Percentages calculated over total RAMIE procedures (n = 478).

Abbreviations: CD indicates Clavien-Dindo grade of postoperative complication.

### Types of Intraoperative Adverse Events

Bleeding was the most frequent event (69.6%), followed by chyle leaks (8.7%), lung injuries (8.7%), airway injuries (4.3%), and other injuries (8.7%) including pericardial, diaphragmatic, colonic serosal, and splenic injuries (Table 1). Most IAEs were classified as grade II (47.8%), followed by grade III (34.8%), grade IV (10.9%), and grade I (6.5%); no grade V events were observed.

### Description and Management of Intraoperative Adverse Events

#### Vascular Injuries

Injuries to aorto-esophageal artery and descending aorta occurred in 11 cases (23.9%) during para-aortic dissection, predominantly in the midthoracic region near the aortic arch, where high-flow branches to the esophagus are frequently encountered. Management relied on compression, followed by targeted placement of a Veriset hemostatic patch (Covidien, Mansfield, MA), as retraction of the injured vessel precluded direct clipping or suturing. Floseal hemostatic sealant (Baxter Corp, Hayward, CA) application was used as an adjunct in most severe cases (see Video 1).

**Video 1:** Aorto-esophageal branch and descending aorta bleeding.mp4.

Azygos vein bleeding occurred in 5 cases (10.9%) during mediastinal pleural or para-aortic dissection using the cautery hook. Most of these injuries were controlled using Hem-o-lok clips (Teleflex Medical, Durham, NC) or 4-0 Prolene sutures.

Injuries to the hepatic artery (3 events, 6.5%) occurred during lymphadenectomy, most commonly along the lateral aspect adjacent to the celiac trunk and were meticulously repaired using 4-0 or 5-0 Prolene sutures. Hepatic perfusion was subsequently assessed; in one case indocyanine green fluorescence imaging was used to confirm arterial patency (see Video 2).

**Video 2:** Hepatic artery bleeding.mp4.

Other bleeding sources accounted for 13 events (28.3%) and included a right gastric artery injury during stapler landing zone preparation, which was repaired with a 4-0 Prolene suture (see Video 3). Remaining bleedings are detailed in Table 1.

**Video 3:** Right gastric artery bleeding.mp4.

#### Chyle Leaks

Chyle leaks occurred in 4 cases (8.7%) and were diagnosed based on visible chylous efflux rather than direct visualization of ductal transection. Following completion of thoracic dissection and lymphadenectomy, leaks were managed by targeted closure with 4-0 Prolene or by mass ligature placement at the expected anatomical location ∼5 cm above the diaphragm (see Video 4). One case required adjunct Hem-o-lok clipping in addition to mass ligature.

**Video 4:** Intraoperative chyle leak management.mp4.

#### Lung Injuries

Lung parenchymal tears occurred in 4 cases (8.7%) during adhesiolysis or during esophageal mobilization and were repaired with 4-0 PDS sutures using either interrupted or running techniques according to defect size; no additional drainage was required.

#### Airway Injuries

Airway injuries involved the left main bronchus, with or without tracheal extension, in 2 cases (4.3%). One was attributed to endotracheal intubation with an oversized tube and high cuff pressure, while the other was identified during tracheoesophageal dissection, without clear distinction between dissection-related or tube-related causation. Both were repaired at the end of the thoracic phase using interrupted 5-0 PDS or Prolene sutures, followed by air leak testing. Repairs were reinforced with an omental patch, with adjunct Veriset in one case. No additional drainage placement was required (see Video 5).

**Video 5:** Injury to the left main bronchus.mp4.

#### Other Intraoperative Adverse Events

Other IAEs included a pericardial injury which was repaired with a 3-0 Vicryl suture; a colonic serosal injury repaired with 3-0 Vicryl suture; and a diaphragmatic defect repaired with interrupted 1-0 Vicryl sutures.

#### Events Requiring Conversion

Most cases with IAEs, 38 of 41 (92.7%), were successfully managed robotically without conversion, allowing completion of the intended procedure. All 3 conversions occurred during the abdominal phase. One followed an inferior vena cava injury during lymphadenectomy of the hepatic artery, requiring conversion for vascular repair in collaboration with the hepatopancreatobiliary team. A second conversion resulted from injury to the left gastric artery at its origin near the celiac trunk during lymphadenectomy. Adequate exposure and safe vascular control could not be achieved robotically. The third conversion occurred after splenic injury during hiatal exposure, where failed hemostatic management with a Veriset patch required conversion and splenectomy.

### Impact of Intraoperative Adverse Events on Perioperative Outcomes

Major IAEs occurred in 20 of 478 patients (4.2%) and were associated with significantly increased mean blood loss (1,211 vs. 296 mL, *P*<0.001), higher conversion rates (15.0% vs. 1.3%, *P*=0.005) and longer mean operative time (478.6±128.2 vs. 413.3±90.4 min, *P*=0.022). Major postoperative complications (35.0% vs. 37.3%, *P*=1.000), median hospital length of stay (11 [10.8–25.0] vs. 11 [8.0–17.0] days, *P*=0.311), and in-hospital mortality (5.0% vs. 2.7%, *P*=0.446) were comparable between groups. Short-term oncologic outcomes were also comparable, with R0 resection achieved in 95.0% vs. 94.5% (*P*=1.000) and a median lymph node yield of 44 [34–54] vs. 40 [26–48] (*P*=0.274). Perioperative outcomes according to IAEs occurrence are shown in Table 2.

**TABLE 2:** Perioperative Outcomes According to the Occurrence of Major Intraoperative Adverse Events

Variable	No IAEs (n=437)	Total IAEs (n=41)	Major IAEs^†^ (n=20)	*P* *
Blood loss, mL (mean±SD)	296.5±203.7	727.7±778.8	1,210.7±977.5	**<0.001**
Operative time, minutes (mean±SD)	413.3±90.4	453.7±109.3	478.6±128.2	**0.022**
Conversion, n (%)	6 (1.4)	3 (7.3)	3 (15.0)	**0.005**
Major postop complication‡, n (%)	163 (37.3)	16 (39.0)	7 (35.0)	1.000
ICU stay, days (median [IQR])	1.0 [1.0–2.0]	1.0 [1.0–4.0]	1.0 [1.0–7.0]	0.353
Length of stay, days (median [IQR])	11.0 [8.0–17.0]	11.0 [8.0–25.0]	11.0 [10.8–25.0]	0.311
In-hospital mortality, n (%)	12 (2.7)	3 (7.3)	1 (5.0)	0.446
R0 resection, n (%)	413 (94.5)	40 (97.6)	19 (95.0)	1.000
Lymph node yield, (median [IQR])	38 [26–49]	38 [26–49]	45 [34–54]	0.269

* *P*-value refers to comparison of major iAE column versus no iAE column.

†Major IAEs were defined as ClassIntra grade III or higher.

𠈁Major postoperative complications were defined as Clavien-Dindo grade III or higher.

Abbreviations: IAEs indicates intraoperative adverse events; ICU, intensive care unit; IQR, interquartile range; SD, standard deviation.

In univariate regression analysis, no association was detected between major IAEs and major postoperative complications (OR 0.895, 95% CI: 0.350–2.288, *P*=0.817). Multivariable logistic regression analysis adjusting for ASA score ≥3 and advanced tumor stage (cT3+ and/or N+), consistently showed no association (aOR 0.907, 95% CI: 0.354–2.324, *P*=0.839). Firth-penalized logistic regression sensitivity analysis yielded similar results (aOR 0.934, 95% CI: 0.366–2.385, *P*=0.887) (see Table 3, Supplemental Digital Content 4, https://links.lww.com/SLA/F918).

## DISCUSSION

This study provides the first comprehensive evaluation of intraoperative adverse events during RAMIE in a high-volume tertiary center. In our consecutive RAMIE cohort, IAEs occurred in 8.6% of patients and were successfully managed robotically in more than 90% of cases without conversion. Major IAEs were associated with increased blood loss, higher conversion rates, and longer operative time. However, major postoperative complication rates (35.0% vs. 37.3%), length of stay, and short-term oncologic outcomes were comparable, with similar R0 resection rates and lymph node yields, in contrast to prior literature on IAEs.^1,2,12^ In-hospital mortality was numerically higher in patients with major IAEs (5.0% vs. 2.7%), although this difference did not reach statistical significance, likely reflecting the limited number of patients with major events. Therefore, these findings should be interpreted with caution, as IAEs still represent a clinically relevant risk to patients.

Previous studies on IAEs during minimally invasive esophagectomy are not specific to robotic surgery and lack standardized definitions.^7^ In a randomized controlled trial, IAEs rates were 16% and 13% for open and robotic esophagectomy, respectively. Two conversions related to intra-abdominal bleeding were reported, while the remaining events were not further characterized.^9^

A recent European multicenter cohort study reported major IAEs in 3.4% of 2,862 patients undergoing thoraco-laparoscopic esophagectomy, comparable to our 4.2% rate.^7^ Although the spectrum of intraoperative injuries was comparable between studies, conversion occurred in 36.7% of eventful cases in their series, whereas we observed a rate of 15.0% when considering major events only.^7^ Postoperative impact of IAEs occurrence was not assessed in that study.

Importantly, all procedures in our cohort were performed by experienced robotic surgeons, while the European multicenter cohort likely included surgeons at different stages of the learning curve, potentially influencing decision-making for conversion.^7^ Whether robotic assistance truly mitigates the need for conversion or whether these differences reflect sample size, case mix, and center experience, remains to be determined. Nevertheless, our low conversion rate in the presence of IAEs aligns with reports from other robotic procedures, such as lung resections, and may reflect technical advantages that facilitate control of complex injuries while preserving a minimally invasive approach.^10^

The evolution of the RAMIE program at our institution provides important context. Following implementation in 2003, early experience showed a conversion rate of 14%,^21^ which decreased to 11% after completion of the learning curve by 2012,^22^ and further declined to 1.9% in the current cohort (2018–2025). This temporal trend reflects how procedural standardization and more than 2 decades of institutional expertise have translated into improved intraoperative control and safety. In this context, the present results may represent a best-case scenario benchmark from a pioneer center and should be interpreted accordingly.

### Strengths and Limitations

This study has several limitations. Underreporting of minor IAEs is possible, as low-grade deviations are often transient and rapidly resolved, limiting their documentation; therefore, the true incidence may be higher than reported. However, analyses of perioperative outcomes were based on major IAEs, which are less susceptible to underreporting and more strongly associated with postoperative outcomes.^1,2,12^ The limited number of major IAEs reduces statistical power for comparative analyses, and a type II error cannot be excluded; the wide confidence intervals around the estimates further reflect this uncertainty and warrant cautious interpretation. The retrospective design, although based on prospectively collected data, introduces the possibility of residual confounding. In addition, long-term oncologic outcomes such as disease-free and overall survival were not assessed. Finally, as a study conducted in a high-volume expert center, the observed incidence and management of these events may not be directly transferable to less experienced settings.

Key strengths include the use of a prospectively maintained database and the application of the validated ClassIntra framework to define and grade IAEs.^1^ Furthermore, routine high-quality video recording enabled detailed analysis of each event,^11^ providing a valuable resource for surgical education by promoting knowledge sharing and dissemination of effective management strategies.^23^

Further research comparing robotic and thoraco-laparoscopic esophagectomy is required to determine whether the technical advantages of the robotic platform translate into improved management of IAEs with lower conversion rates, while also clarifying their impact on long-term oncologic outcomes such as recurrence and survival.

To our knowledge, this is the first study to prospectively capture, classify, and analyze IAEs in RAMIE using a validated framework, while also detailing their technical management through systematic video review.

## CONCLUSION

Our results demonstrated that intraoperative adverse events during RAMIE are rare and can safely be managed robotically without conversion in more than 90% of cases. In this expert-center cohort, major IAEs were not associated with a statistically significant increase in short-term postoperative morbidity, although this finding requires confirmation in larger multicenter studies due to the limited number of events.

## Supplementary Material

## DISCUSSANTS

### Simon Y.K. Law (Hong Kong, China)

First, I would like to congratulate the investigators on an excellent paper and results from RAMIE in a high-volume center. As you’ve already pointed out, intraoperative complications occurred in about 8% of patients. Major complications occurred at 4%. And I think your main conclusion is that most complications can be managed robotically. Although it affects perioperative outcomes, such as operating time, postoperative complications were not affected. With regard to the strengths and drawbacks of the study, you’ve already pointed out most of them. I would just like to ask you 3 questions. Although the series focuses on robotic esophagectomy, I guess you’ve also done a lot of VATS and laparoscopic esophagectomy as well. In your data, could you manage all these complications using VATS or laparoscopically before the era of robotic surgery? Although RAMIE is obviously a great technique, can you have very similar results just using VATS and laparoscopic methods? Around the world, not everyone has a robot. And sometimes even in hospitals with robots, robotic console time is limited. There are still many VATS and laparoscopic esophagectomies around the world. And hopefully, we can manage these complications similarly. The second question I have is that I noted that 123 patients underwent transhiatal esophagectomy. I’m wondering if complications occur, is it as easy to manage robotically? Because you have very limited space, and exposure may be suboptimal. You didn’t detail those complications in your paper. What are your thoughts on managing complications when you’re doing a transhiatal esophagectomy? Increasingly, it’s going to be more relevant because, as you know, in Japan, in China, and in some centers around the world now, they’re doing transmediastinal esophagectomy, whether by just conventional laparoscopic instruments or by robotic assistance. Complications are going to occur, and it would be quite difficult to manage transmediastinally. The last question is that you have got excellent results, in a very experienced center. But would that give other less experienced centers a false sense of security, thinking that they can do the same? And so, delay conversion and lead to an unfavorable outcome? Do you have any views on that?

### Response From Franco Badaloni (Utrecht, The Netherlands)

Thank you very much for your comments on our manuscript. We have no data regarding intraoperative adverse events during conventional minimally invasive esophagectomy in our center, and this comparison would not be entirely fair, as our center’s expertise is primarily focused on the robotic approach. That said, we believe there are at least two reasons why robotic assistance may offer an advantage. First, we have autonomy. The surgeon is in control of all instruments, including the camera and a third arm, and therefore does not have to rely on the performance of the team as much as in thoraco-laparoscopic surgery. Second, the robot gives us the possibility of achieving technical control with detailed 5-0 sutures in a confined thoracic space that requires a level of precision and dexterity that conventional laparoscopy simply cannot replicate, regardless of surgeon experience.

Regarding the second question, fortunately we did not encounter any mediastinal bleeding or injuries during transhiatal approaches, as all five events in this group occurred during the abdominal phase. In the hypothetical scenario of a mediastinal injury during the transhiatal approach, we believe the optimal management would depend on the type of complication. If the patient is unstable because of bleeding, conversion to open would probably be the most logical thing to do, as docking of the robot would take additional time. However, in the case of an airway injury, where there is sufficient time for repair, our view is the opposite: the robotic platform would be our preferred approach.

The final point is extremely important. Our results should not be interpreted as a green light for less experienced centers to attempt robotic management of major intraoperative adverse events without expertise. The key point here is that the surgical team must know its own expertise. Not what others are doing, not what the literature reports. That said, we do want to encourage robotic surgeons to give the platform a chance before converting. A surgeon who has already completed the learning curve in robotic esophagectomy has developed robotic technical skills that may be useful to manage these complications.

### Lorenzo E. Ferri Lorenzo (Montreal, Canada)

Wonderful presentation, and I applaud you on presenting something that’s uncomfortable for all of us in this room. But I will caution everyone in this room that it’s important that we take ownership of this, because I’ve seen so many centers shut down because we don’t acknowledge and recognize our complications, and we have a framework to address them. Also, I applaud the incredible results of the extremely low conversion rate, which we must recognize conversion is not a failure, but it’s still conversion, nonetheless. I applaud you for the incredible outcome. The question I have is: do you think any of the complications you had were specific to robotics? Is there something that you’re missing in haptics that make it more likely? And the one I’m thinking of, because every one of my fellows has, is to end up having to do a splenectomy once they cut right through the splenic artery doing a D2 dissection using robotic. So, do you think there’s any complications that you found specific to the robot due to the lack of haptics?

### Response From Franco Badaloni (Utrecht, The Netherlands)

Thank you very much for your comment. It would certainly be very interesting to identify which intraoperative adverse events were specifically related to the robotic platform. However, after reviewing the complications, I believe that most of them could also occur during conventional minimally invasive surgery, regardless of the lack of haptic feedback. We had one transverse mesocolon injury that occurred when one of the instruments was advanced outside the field of view. That is something that could also happen in conventional minimally invasive surgery. The splenic injury may be a better example. It occurred while placing the vessel sealer in a way to expose the hiatus, and perhaps with haptic feedback, the surgeon might have realized earlier that excessive traction was being applied. Overall, I would say that two events could reasonably be attributed partially to the lack of haptic feedback.

### Zhouqiao Wu (Bejing, China)

I have a question that I think is quite important, as it may lead to different interpretations of your findings. For patients who experienced intraoperative adverse events, you reported that these events were not associated with an increase in postoperative complications. My question is whether this outcome reflects effective intraoperative management, after which patients were treated according to routine postoperative care pathways, or whether the occurrence of an intraoperative event led the surgical team to pay closer attention postoperatively, with more intensive monitoring or additional examinations that may have helped prevent subsequent complications. These two possibilities would lead to different interpretations of your results. The first would suggest that once the intraoperative event is appropriately managed, routine postoperative care may be sufficient. The second would imply that enhanced postoperative surveillance is necessary for these patients.

Given that the event cohort appears to be relatively small, I suggest further exploring this issue and, if possible, providing additional information on postoperative management and monitoring in this subgroup.

### Response From Franco Badaloni (Utrecht, The Netherlands)

That’s an interesting question. Our postoperative management did not change based on whether an intraoperative adverse event occurred, except in cases of airway injuries, where we place different chest drains intraoperatively. Otherwise, our postoperative protocol was exactly the same for all patients. However, it would be interesting to explore whether postoperative management should be tailored according to the occurrence, type, and severity of intraoperative adverse events. For example, after severe intraoperative bleeding, we may pay closer attention to these patients or even consider a more specific postoperative protocol for them.
