# Supplementary material for: Intraoperative Adverse Events in Robot-Assisted Minimally Invasive Esophagectomy (RAMIE): Incidence, Management, and Impact on Surgical Outcomes
Source: Ann Surg. 2026 Aug 19;284(5):957–63. doi: 10.1097/SLA.0000000000007167 (PMC13652120; doi:10.1097/SLA.0000000000007167)
Supplement: Supplementary file 1 [file sla-284-0957-s006.docx]

**Supplemental Digital Content 1**

**Table 1.** ClassIntra version 1.0 classification of intraoperative adverse events [1].

| **Grade** | **Definition** | **Examples** |
| --- | --- | --- |
| **Grade 0** | No deviation from the ideal intraoperative course | - |
| **Grade I** | Any deviation from the ideal intraoperative course:  • Without the need for any additional treatment or intervention  • Patient with no or mild symptoms | • Bleeding: bleeding above average from small caliber vessel, self-limiting or definitively manageable without additional treatment than routine coagulation  • Injury: minimal serosal intestinal lesion, not requiring any additional treatment  • Cautery: small burn of the skin, no treatment necessary  • Arrhythmia: arrhythmia (eg, extrasystoles) without relevance |
| **Grade II** | Any deviation from the ideal intraoperative course:  • With the need for any additional minor treatment or intervention  • Patient with moderate symptoms, not life threatening, and not leading to permanent disability | • Bleeding: bleeding from medium caliber artery or vein, ligation; use of tranexamic acid  • Injury: non-transmural intestinal lesion requiring suture(s)  • Cautery: moderate burn requiring non-invasive wound care  • Arrhythmia: arrhythmia requiring administration of antiarrhythmic drug, no hemodynamic effect |
| **Grade III** | Any deviation from the ideal intraoperative course:  • With the need for any additional moderate treatment or intervention  • Patient with severe symptoms, potentially life threatening or potentially leading to permanent disability | • Bleeding: bleeding from large caliber artery or vein with transient hemodynamic instability, ligation or suture; blood transfusion  • Injury: transmural intestinal lesion requiring segmental resection  • Cautery: severe burn requiring surgical debridement  • Arrhythmia: arrhythmia requiring administration of antiarrhythmic drug, transient hemodynamic effect |
| **Grade IV** | Any deviation from the ideal intraoperative course:  • With the need for any additional major and urgent treatment or intervention  • Patient with life threatening symptoms or leading to permanent disability | • Bleeding: life threatening bleeding with splenectomy; massive blood transfusion; stay at intensive care unit  • Injury: injury of central artery or vein requiring extended intestinal resection  • Cautery: life threatening burn injury by cautery leading to fire requiring intensive care treatment  • Arrhythmia: arrhythmia requiring electroconversion, defibrillation, or admission to intensive care |
| **Grade V** | Any deviation from the ideal intraoperative course with intraoperative death of the patient | - |

The classification defines intraoperative adverse events as any deviation from the ideal intraoperative course occurring between skin incision and skin closure. Any event related to surgery and anesthesia during the index surgery must be considered and should be rated directly after surgery (these events were not defined as intraoperative adverse events: sequelae, failures of cure, events related to the underlying disease, incorrect site or incorrect patient surgery, or errors in indication). A requirement is that the indication for surgery and the interventions conform to current guidelines.
