# Supplementary material for: Intraoperative Adverse Events in Robot-Assisted Minimally Invasive Esophagectomy (RAMIE): Incidence, Management, and Impact on Surgical Outcomes
Source: Ann Surg. 2026 Aug 19;284(5):957–63. doi: 10.1097/SLA.0000000000007167 (PMC13652120; doi:10.1097/SLA.0000000000007167)
Supplement: Supplementary file 2 [file sla-284-0957-s007.docx]

**Table 2.** Baseline characteristics according to the occurrence of intraoperative adverse events.

| **Variable** | **Total**  (n = 478) | **No IAEs**  (n = 437) | **Total IAEs**  (n = 41) | **Major IAEs**  (n = 20) | **p-value*** |
| --- | --- | --- | --- | --- | --- |
| **Age, years (mean ± SD)** | 66.8 ± 8.6 | 66.9 ± 8.7 | 65.9 ± 7.6 | 66.1 ± 7.6 | 0.492 |
| **Male sex, n (%)** | 356 (74.5%) | 328 (75.1%) | 28 (68.3%) | 14 (70.0%) | 0.806 |
| **BMI, kg/m² (median [IQR])** | 25.9 [23.3–28.1] | 25.9 [23.4–28.1] | 25.2 [22.6–27.5] | 24.9 [22.8–26.9] | 0.295 |
| **ASA score, n (%)** |  |  |  |  | 0.379 |
| I | 32 (6.7%) | 32 (7.3%) | 0 (0.0%) | 0 (0.0%) |  |
| II | 242 (50.6%) | 216 (49.4%) | 26 (63.4%) | 13 (65.0%) |  |
| III | 192 (40.2%) | 177 (40.5%) | 15 (36.6%) | 7 (35.0%) |  |
| IV | 12 (2.5%) | 12 (2.7%) | 0 (0.0%) | 0 (0.0%) |  |
| **Comorbidities, n (%)** |  |  |  |  |  |
| Any comorbidity | 339 (70.9%) | 315 (72.1%) | 24 (58.5%) | 12 (60.0%) | 0.359 |
| Cardiac | 117 (24.5%) | 111 (25.4%) | 6 (14.6%) | 3 (15.0%) | 0.429 |
| Vascular | 187 (39.1%) | 175 (40.0%) | 12 (29.3%) | 6 (30.0%) | 0.506 |
| Diabetes mellitus | 79 (16.5%) | 75 (17.2%) | 4 (9.8%) | 2 (10.0%) | 0.550 |
| Pulmonary | 85 (17.8%) | 77 (17.6%) | 8 (19.5%) | 3 (15.0%) | 1.000 |
| Neurological | 54 (11.3%) | 50 (11.4%) | 4 (9.8%) | 2 (10.0%) | 1.000 |
| **Clinical T stage, n (%)** |  |  |  |  | 0.859 |
| Tis | 1 (0.2%) | 1 (0.2%) | 0 (0.0%) | 0 (0.0%) |  |
| T1 | 19 (4.0%) | 18 (4.1%) | 1 (2.4%) | 0 (0.0%) |  |
| T2 | 94 (19.7%) | 86 (19.7%) | 8 (19.5%) | 5 (25.0%) |  |
| T3 | 305 (63.8%) | 279 (63.8%) | 26 (63.4%) | 12 (60.0%) |  |
| T4 | 59 (12.3%) | 53 (12.1%) | 6 (14.6%) | 3 (15.0%) |  |
| **Clinical N stage, n (%)** |  |  |  |  | 0.490 |
| N0 | 223 (46.7%) | 205 (46.9%) | 18 (43.9%) | 11 (55.0%) |  |
| N1 | 166 (34.7%) | 153 (35.0%) | 13 (31.7%) | 5 (25.0%) |  |
| N2 | 65 (13.6%) | 58 (13.3%) | 7 (17.1%) | 4 (20.0%) |  |
| N3 | 24 (5.0%) | 21 (4.8%) | 3 (7.3%) | 0 (0.0%) |  |
| **Neoadjuvant therapy, n (%)** |  |  |  |  | 1.000 |
| Yes | 411 (86.0%) | 374 (85.6%) | 37 (90.2%) | 17 (85.0%) |  |
| No | 67 (14.0%) | 63 (14.4%) | 4 (9.8%) | 3 (15.0%) |  |
| **Operative technique, n (%)** |  |  |  |  | 0.778 |
| Transhiatal | 123 (25.7%) | 118 (27.0%) | 5 (12.2%) | 4 (20.0%) |  |
| Ivor Lewis (intrathoracic) | 228 (47.7%) | 205 (56.1%) | 23 (56.1%) | 10 (50.0%) |  |
| McKeown (cervical) | 127 (26.6%) | 114 (26.1%) | 13 (31.7%) | 5 (30.0%) |  |

* P -value refers to comparison of Major iAE column versus no iAE column.
Abbreviations: IAEs, intraoperative adverse events; BMI, body mass index.
