# Supplementary material for: Intraoperative Adverse Events in Robot-Assisted Minimally Invasive Esophagectomy (RAMIE): Incidence, Management, and Impact on Surgical Outcomes
Source: Ann Surg. 2026 Aug 19;284(5):957–63. doi: 10.1097/SLA.0000000000007167 (PMC13652120; doi:10.1097/SLA.0000000000007167)
Supplement: Supplementary file 3 [file sla-284-0957-s008.docx]

**Supplemental Digital Content 4**

**Table 3**. Univariate, multivariate, and Firth sensitivity logistic regression analysis for major postoperative complications.

|  | **Univariate** | | **Multivariate** | | **Firth Sensitivity Analysis*** | |
| --- | --- | --- | --- | --- | --- | --- |
| **Variable** | **OR  (95% CI)** | **p- value** | **aOR  (95% CI)** | **p- value** | **aOR  (95% CI)** | **p- value** |
| **Major IAEs** | 0.895  (0.350–2.288) | 0.817 | 0.907  (0.354–2.324) | 0.839 | 0.934  (0.366–2.385) | 0.887 |
| **ASA score ≥ 3** | 1.272  (0.875–1.848) | 0.207 | 1.266  (0.871–1.840) | 0.217 | 1.264  (0.870–1.837) | 0.220 |
| **Advanced tumor stage**  (cT3+ and/or N+) | 0.806  (0.499–1.303) | 0.379 | 0.811  (0.502–1.312) | 0.349 | 0.810  (0.501–1.310) | 0.390 |

*Firth: Firth penalized logistic regression analysis.

Abbreviations: IAEs, intraoperative adverse events; OR, odds ratio; CI, confidence interval; aOR, adjusted odds ratio.
