# Supplementary material for: Intraoperative Adverse Events in Robot-Assisted Minimally Invasive Esophagectomy (RAMIE): Incidence, Management, and Impact on Surgical Outcomes
Source: Ann Surg. 2026 Aug 19;284(5):957–63. doi: 10.1097/SLA.0000000000007167 (PMC13652120; doi:10.1097/SLA.0000000000007167)
Supplement: Supplementary file 4 [file sla-284-0957-s009.docx]

**Figure 1.** Patient flowchart and analytical framework for intraoperative adverse events during RAMIE.
